# Characterization of genetic determinants of the resistance to phylloxera, *Daktulosphaira vitifoliae,* and the dagger nematode *Xiphinema index* from muscadine background

**DOI:** 10.1186/s12870-020-2310-0

**Published:** 2020-05-12

**Authors:** Bernadette Rubio, Guillaume Lalanne-Tisné, Roger Voisin, Jean-Pascal Tandonnet, Ulysse Portier, Cyril Van Ghelder, Maria Lafargue, Jean-Pierre Petit, Martine Donnart, Benjamin Joubard, Pierre-François Bert, Daciana Papura, Loïc Le Cunff, Nathalie Ollat, Daniel Esmenjaud

**Affiliations:** 1 INRAE, UMR EGFV, 33883 Villenave d’Ornon, France; 2 IFV, Domaine de l’Espiguette, 30240 Le Grau du Roi, France; 3 INRAE, Université Nice Côte d’Azur, CNRS, ISA, 06903 Sophia Antipolis, France; 4 INRAE, UMR SAVE, 33883 Villenave d’Ornon, France

**Keywords:** Grapevine, Rootstock, Phylloxera, Nematode, Genetic architecture, Pest resistance, Muscadine

## Abstract

**Background:**

Muscadine (*Muscadinia rotundifolia*) is known as a resistance source to many pests and diseases in grapevine. The genetics of its resistance to two major grapevine pests, the phylloxera *D. vitifoliae* and the dagger nematode *X. index*, vector of the *Grapevine fanleaf virus* (GFLV), was investigated in a backcross progeny between the F1 resistant hybrid material VRH8771 (*Vitis-Muscadinia*) derived from the muscadine R source ‘NC184–4’ and *V. vinifera* cv. ‘Cabernet-Sauvignon’ (CS).

**Results:**

In this pseudo-testcross, parental maps were constructed using simple-sequence repeats markers and single nucleotide polymorphism markers from a GBS approach. For the VRH8771 map, 2271 SNP and 135 SSR markers were assembled, resulting in 19 linkage groups (LG) and an average distance between markers of 0.98 cM. Phylloxera resistance was assessed by monitoring root nodosity number in an *in planta* experiment and larval development in a root in vitro assay. Nematode resistance was studied using 10–12 month long tests for the selection of durable resistance and rating criteria based on nematode reproduction factor and gall index. A major QTL for phylloxera larval development, explaining more than 70% of the total variance and co-localizing with a QTL for nodosity number, was identified on LG 7 and designated *RDV6*. Additional QTLs were detected on LG 3 (*RDV7*) and LG 10 (*RDV8*), depending on the *in planta* or in vitro experiments, suggesting that various loci may influence or modulate nodosity formation and larval development. Using a Bulked Segregant Analysis approach and a proportion test, markers clustered in three regions on LG 9, LG 10 and LG 18 were shown to be associated to the nematode resistant phenotype. QTL analysis confirmed the results and QTLs were thus designated respectively *XiR2, XiR3 and XiR4*, although a LOD-score below the significant threshold value was obtained for the QTL on LG 18.

**Conclusions:**

Based on a high-resolution linkage map and a segregating grapevine backcross progeny, the first QTLs for resistance to *D. vitifoliae* and to *X. index* were identified from a muscadine source. All together these results open the way to the development of marker-assisted selection in grapevine rootstock breeding programs based on muscadine derived resistance to phylloxera and to *X. index* in order to delay GFLV transmission.

## Background

Domesticated grapevine, *Vitis vinifera* sub-sp. *vinifera* [1], is grown grafted in most countries worldwide. Grafting has been successfully used to cope mainly with phylloxera (*Daktulosphaira vitifoliae*), an insect-pest that destroyed the European vineyard after its introduction from North America in the middle of the nineteenth century [2, 3] and participate to the control of other soil-borne pests and adaptation to abiotic stresses.

In addition to Europe, grapevine phylloxera spread quickly to most wine grape growing regions of the world including South Africa, Middle East, Asia and Australia [4]. This insect has two different forms, the radicicoles and the gallicoles, which affect roots and leaves, respectively. Radicicoles are the most destructive in *V. vinifera* due to severe root damage, while gallicole forms are more common in most other species without lethal effects for the plants. Root feeding induces the formation of galls called nodosities [4, 5]. Phylloxera root infection causes vine decline and finally plant death, partly explained by secondary fungal infections [2]. The use of resistant rootstocks from *Vitis* species other than *V. vinifera* is advocated as the main method of radicicole phylloxera management and may be considered as the most sustainable example of biological control for a pest ever used [6]. Several sources of grape phylloxera resistance for rootstock breeding have been identified. Heritability studies have shown that a variable number of loci control the resistance trait [7–11]. The first genetic mapping of a QTL for phylloxera resistance identified the *RDV1 (RESISTANCE DAKTULOSPHAIRA VITIFOLIAE 1)* locus on chromosome 13 in the Börner (*V. riparia x V. cinerea*) rootstock [12]. The *RESISTANCE DAKTULOSPHAIRA VITIFOLIAE 2 (RDV2)* locus was mapped to chromosome 14 in *V. cinerea* cv. ‘C2–50’ [5]. A leaf-specific phylloxera resistance locus overlapped the location of *RDV2* on LG 14 in a F1 family from a cross containing at least six *Vitis* species in the ancestry (*V. vinifera, V. riparia, V. rupestris, V. labrusca, V. aestivalis* and *V. berlandieri*). In contrast, from the same plant material, a locus for resistance to the root form of phylloxera mapped to chromosomes 5 and 10 [13].

In addition to phylloxera, rootstocks may contribute to the control of other soil-borne pests such as root-knot and dagger nematodes. Resistance to the dagger nematode *Xiphinema index* is an important objective in grape rootstock breeding programs. *X. index* is a migratory ectoparasite that primarily feeds on the root tips of grapevines and causes severe damage to their root system. More significantly, *X. index* is recognized as the vector of *Grapevine fanleaf virus* (GFLV), the causal agent of the fanleaf degeneration disease which is considered to be one of the major threats to the grapevine industry [14, 15]. *X. index* can survive in soils of ancient vineyards and retain GFLV for many years without the presence of host plant [16]. Nematicides and fumigants failed to control the dagger nematode because of their poor penetration in deep soil layers where *X. index* mainly survives [17, 18] and their use is now banned in most countries because of their high toxicity. In order to overcome these limitations, using nematode-resistant grapevine rootstocks appears as one of the most promising control method to significantly delay viral transmission. The highest level of resistance to *X. index* was found in *V. arizonica, V. candicans, V. rufotomentosa, V. smalliana* and *V. solonis* [19, 20]. Resistance in *V. arizonica* is controlled by a single major locus, *XIPHINEMA INDEX RESISTANCE 1 (XiR1)* located on chromosome 19 [21, 22].

In the next decades, grape growing will have to face several challenges, such as climate change, decrease in pesticides application, water availability and competition for arable lands with food crops. In this context, it is important to consider the rootstock as a key component of adaptation and consequently, breeding new rootstocks is urgently needed. Rootstock breeding programs conducted throughout the world share similar goals, such as resistance to phylloxera and nematodes (dagger and root-knot nematodes), vigour management, mineral uptake efficiency, together with drought, salinity and limestone tolerances [20, 23]. The world’s existing rootstocks have a very narrow genetic base derived from only a few American grape species accessions [24]. Despite the diversity available among the genus *Vitis*, only 4 to 5 species are commonly used in crosses, i.e. *V. berlandieri*, *V. riparia*, *V. rupestris*, *V. champini* and *V. vinifera*. *Muscadinia rotundifolia*, the muscadine vine, was also considered as a highly interesting species for rootstock improvement because of its resistance to many pests and diseases [10]. Thus, hybrids between this American species and *V. vinifera* were created in order to be used as rootstocks [25] or as parents or elite materials for further crosses [26]. However, muscadines present major defaults such as poor rooting, grafting incompatibility or mineral deficiencies, advanced crosses are necessary to breed highly and performing rootstocks.

Grapevine breeding takes generally decades to release an advanced cultivar. Considering the difficulty to screen for most traits of interest in rootstocks, the development of molecular-assisted selection is a priority. This relies on the identification of one or few loci closely associated to the trait expression and explaining a high percentage of phenotypic variance. As previously mentioned, only four loci have been identified so far in response to phylloxera in various *Vitis* species and a single one in response to *X. index*. Therefore it is crucial to improve our knowledge about the resistance response of *Vitis* and *Muscadinia* spp. resources to major grapevine pests and to analyse their genetic determinism. Recent progress in the development and application of molecular markers, genetic mapping and whole genome sequencing combined with high throughput technologies will help to better characterize the genetic bases of the traits of interest [27]. For grapevine, the development and application of SSR (Simple Sequence Repeat) markers are considered as a key step of the construction of molecular maps [21, 28, 29]. Next-generation sequencing (NGS) has then facilitated the development of methods to genotype very large numbers of SNP (Single Nucleotide Polymorphism) markers [30, 31]. Genotyping-by-sequencing (GBS) has been developed as a rapid and robust approach to sequencing of multiplexed samples [32, 33]. The work by Barba and collaborators [34] demonstrated the first application of the GBS procedure in generating SNPs to construct a high resolution map for QTL mapping in grapevine.

In order to identify additional sources of resistance to the phylloxera and to the dagger nematode, a backcross 1 (BC1) population from the cross between a muscadine hybrid (VRH8771 = *V. vinifera x M. rotundifolia*) and *V. vinifera* cv. ‘Cabernet-Sauvignon’ has been characterized in this study. Through this cross, one seeks to expand the existing knowledge about the genetic determinism of *M. rotundifolia* natural resistances towards the pathogens and pests. The main objectives of our study were i) to build SSR and SNP-based linkage maps using pseudo-testcross strategy and ii) to exploit them for QTL mapping of the genetic bases of the resistance responses to these major grapevine pests. These goals are the first steps toward the development of marker-assisted breeding for resistance to phylloxera and *X. index*.

## Results

### Genotyping: sequencing, SNP calling and SNP selection

A total of 84 million cleaned reads were obtained, and the number of reads per sample ranged from 36,000 to 9.5 million, with an average of 3.05 million. This number of reads was equivalent to ~ 0.91-fold coverage of other *Vitis* genomes which are estimated to have a size of approximately 500 Mb. The SNP calling performed with GATK pipeline and VCFtools produced a total of 324,183 SNPs among which 52,625 have been identified as pseudotestcross markers. By excluding sites based on missing data or segregation distortion, two sets of 2285 and 738 SNPs were obtained for the female (VRH8771) and the male (CS) maps, respectively. These sets were complemented by 135 and 148 SSRs genotyped on 90 BC1 individuals for the construction of the female and male maps, respectively. Because of the missing data threshold per individual and the genotype frequencies expected, the final dataset included 92 BC1 individuals. In the end, 75 BC1 individuals were genotyped with both SSRs and SNPs markers, 19 BC1 individuals with SNPs markers only and 17 BC1 individuals with SSRs markers only (Table S1).

### High density maternal and paternal genetic maps

Maternal (VRH8771) and paternal (CS) maps were constructed using 2420 and 886 sets of markers (SSR and SNP markers), respectively (Fig. S1 and Fig. S2). For each set of markers, 19 LGs were produced and the final sizes for the VRH8771 and CS maps were 2351 and 1982 cM, respectively. The map density or average distance between markers for VRH8771 and CS maps was 0.98 and 2.24 cM, respectively (Table 1). The comparison of marker order with their physical position in VRH8771 and CS maps showed that most plots were on the diagonal or adjacent position (Fig. S3).

**Table 1:**
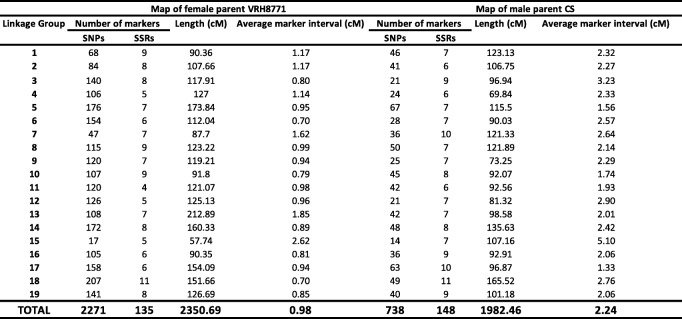
Characteristics of the maternal (VRH8771) and the paternal (*V. vinifera* cv. ‘Cabernet-Sauvignon’, CS) genetic maps of the BC1 cross VRH8771 x CS

### Phenotypic evaluation of resistance to *D. vitifoliae*

**Fig. 1:**
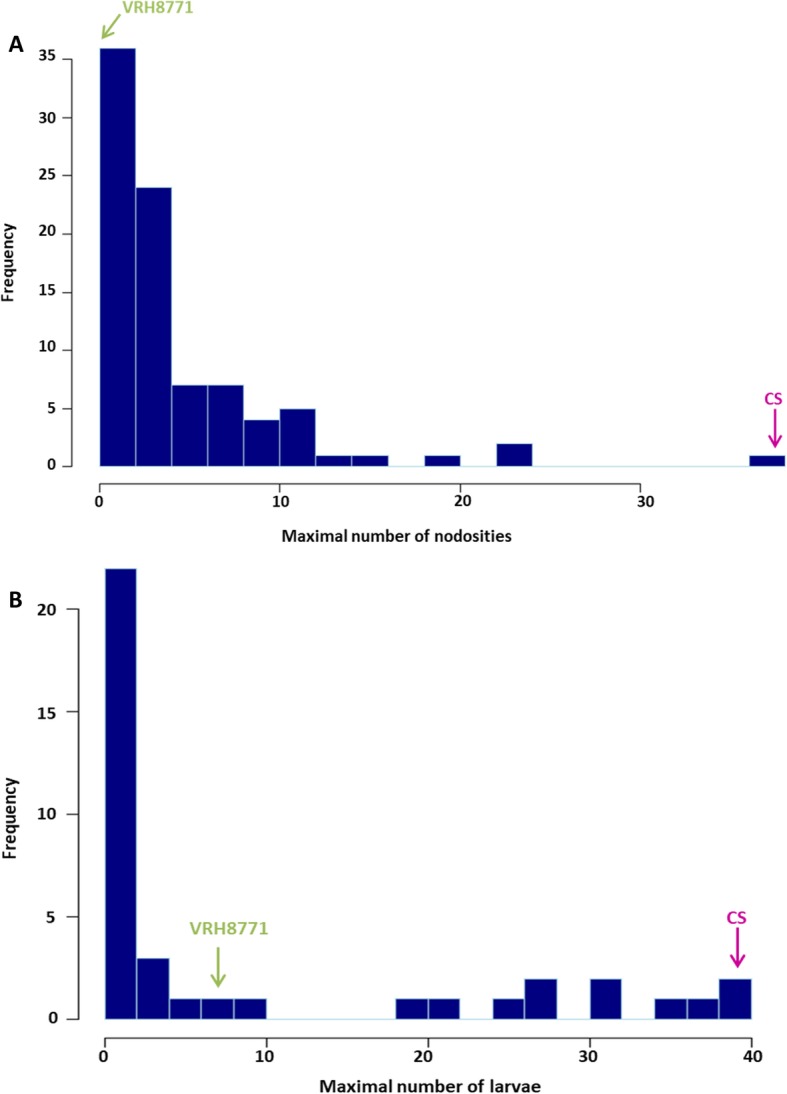
Distribution of the BC1 individuals according to (**a**) the maximal number of nodosities (*in planta* experiment) and (**b**) the maximal number of larvae (in vitro experiment). Parental genotypes, VRH8771 and CS, are represented by a green and pink bar, respectively

The phenotypic scores of phylloxera root resistance were obtained in both *in planta* and in vitro experiments using 89 and 37 BC1 individuals, respectively. In the *in planta* experiment, results were expressed by a number of nodosities per plant whereas in the in vitro experiment they corresponded to a number of larvae counted on the five root pieces of each individual. A Spearman correlation between in vitro and *in planta* data was performed revealing a positive and significant correlation (r = 0.47, *p-value* = 0.003). Because of the effects of inoculation conditions, there was a large variability for nodosity and larvae numbers among the three replicates of each BC1 individual plant tested, as illustrated by an average value of 73 and 53% for the variation coefficients of *in planta* and in vitro experiments, respectively. Therefore, the maximal numbers of nodosities and larvae per individual were confirmed to be reliable indicators of the quantitative resistance phenotype [12]. The negative control genotype, ‘Börner’, exhibited the expected results with a maximum number of 2 nodosities *in planta* and 9 larvae in vitro, respectively. Phenotypic responses of both parental genotypes were in agreement with what was expected, as VRH8771 resulted resistant and CS susceptible (Fig. 1). Over the population, the number of nodosities ranged from 0 to 37 (Fig. 1a) and the number of larvae ranged from 0 to 39 (Fig. 1b) in *in planta* and in vitro experiments, respectively. In both experiments, there were extensive variations of the phylloxera responses, with values of broad-sense heritabilities being 0.48 and 0.74 for nodosity and larvae numbers, respectively.

### Phenotypic evaluation of resistance to *X. index*

**Fig. 2:**
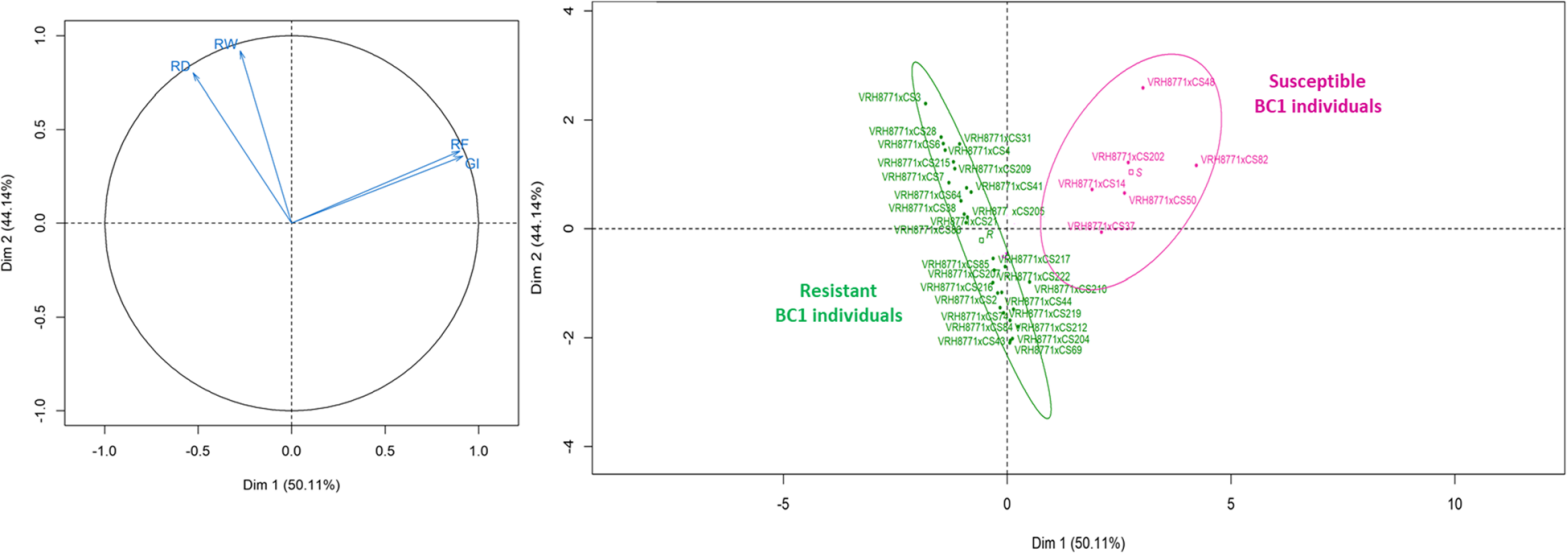
Principal component analysis (PCA) of the 35 BC1 individuals tested in 2010–2011 and 2011–2012 experiments with root system development (RD), root weight (RW), nematode reproduction factor (RF) and gall index (GI). The two major principal components that accounted for 94.25% of the variance have been plotted. The rating criteria factor map (left) and the individuals factor map (right) are reported. In the individual factor map, the BC1 individuals were assigned to two distinct groups, the resistant group in green and the susceptible group in pink, based on their RF and GI criteria

In the first two experiments (2010–2011 and 2011–2012), resistance to *X. index* was screened from 35 BC1 individuals, using roots characteristics (root development (RD) and root weight (RW)), the nematode reproduction factor (RF) and the gall index (GI). The data obtained from these two experiments were analyzed together since no year effect was identified (Fig. S4 and Table S2). A principal component analysis (PCA) using those 35 individuals and the RD, RW, RF and GI criteria showed that the two first axes explained 94.25% of total variation (Fig. 2). Based on both the RF and GI criteria, resistant (R) and susceptible (S) groups were clearly identified. These two groups were separated along the first dimension of the PCA which is represented by these nematode development criteria. PCA demonstrated that susceptibility criteria vary independently from root characteristics. Statistical analyzes confirmed this result with significant differences in GI and RF values between resistant and susceptible BC1 individuals (Wilcoxon signed-rank test – *p-values* of 0.00015 and 0.00013 for RF and GI, respectively). On the other hand, no statistically significant differences were revealed between resistant and susceptible BC1 individuals for the criteria (RD and RW) related to the roots characteristics (Wilcoxon signed-rank test – *p-values* of 0.335 and 0.535 for RD and GW, respectively).

Spearman correlations were calculated among RD, RW, RF and GI criteria. There was no significant correlation between RD or RW on the one hand and RF or GI on the other hand, which confirmed the independence of the root and nematode developmental characteristics. The nematode reproduction factor (RF) was highly correlated with the gall index (GI) (r = 0.59, *p-value* = 0.00019) (Table S3). Such a high significant correlation made it possible to rely on the gall index rating in order to score the response of the 60 total BC1 individuals to *X. index* within the independent experiments. An individual is classified as resistant when its replicates have a mean GI value lower than 1 and as susceptible when its replicates have a mean GI value equal or above 1. Thus, on all five experiments, there were 50 and 10 BC1 individuals characterized as resistant and susceptible, respectively. This segregation ratio 50R:10S fits the ratio of 7:1 (χ^2^_0.05_ = 0.5978) expected when three dominant and independent genes control the resistance.

### Genetic determinism and mapping of the response to *D. vitifoliae*

QTLs were detected for all traits scored and for their associated BLUP values in *in planta* and in vitro experiments. On the high-density genetic map of VRH8771 (Table 2), seven total QTLs were identified for both the maximum numbers of nodosities and the maximum numbers of larvae. These QTLs were localized on three LGs i.e. LG 3, LG 7 and LG 10. LOD scores and the explained phenotypic variances had higher values under in vitro conditions. This might be due to the lower environmental variations occurring under in vitro than under greenhouse conditions. On LG 7, several QTLs were found whatever the conditions and criteria within a confidence interval of 60 cM. In this group, the two QTLs detected for the criteria ‘maximum number of nodosities’ and ‘BLUP nodosities’ in *in planta* experiment explained each approximately 20% of the variability while the two QTLs detected in in vitro conditions for the criteria ‘number of larvae’ and ‘BLUP larvae’ explained variances of 87 and 70%, respectively. Two QTLs were identified on LG 10 in *in planta* and in vitro experiments, respectively with the maximal number of nodosities and the BLUP values associated to the maximal number of larvae. Finally, analysis of BLUP values associated to the maximal number of nodosities in *in planta* conditions, showed a QTL on LG 3.

**Table 2:**
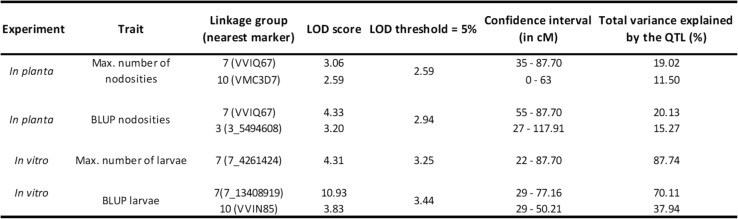
Location and characteristics of the QTLs identified in *in planta* and in vitro experiments in response to *D. vitifoliae*

### Genetic determinism and mapping of the response to *X. index*

The 7R:1S segregation ratio observed in our tests might suggest the presence of three dominant and independent resistance (R) factors controlling the response to *X. index*. With this hypothesis in mind for the detection of markers linked to resistance, we first used a method derived from the bulked-segregant analysis (BSA) [35]. As a double pseudo-testcross strategy was developed in the construction of the genetic maps, access to information on the different allelic forms was available only for SSR markers and, in a first step, the BSA-type analysis was performed using only these markers. From the 2 (ab), 3 (abc) or 4 (abcd) putative allelic forms of SSRs, we retained the markers for which an allele was detected in the resistant parent (VRH8771) and in a part of the resistant BC1 individuals but was lacking in all the susceptible BC1 individuals and the susceptible parent (CS). Markers meeting this requirement were located on the three linkage groups LG 9, LG 10 and LG 18 (Table 3), which is in line with the hypothesis of three dominant and independent R factors.

**Table 3:**
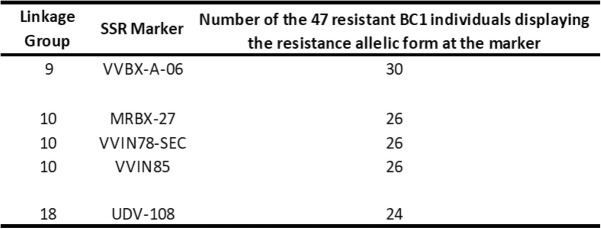
Distribution of the resistance alleles identified in the resistant parent VRH8771 using SSRs markers among resistant BC1 individuals in response to *X. index*

In order to use all the available genetic information related to the SSR and SNP markers, a proportion test was performed. This test aimed at comparing the proportion of resistant and susceptible individuals at each marker. Markers for which a statistically significant difference between resistant and susceptible proportions have been obtained, have been considered as potentially involved in the *X. index* resistance response. Such groups of markers with significant differences in their R/S proportions were found on LG 9, LG 10 and LG 18, corroborating the results obtained by BSA-type analyses from SSR markers (Table S4).

**Fig. 3:**
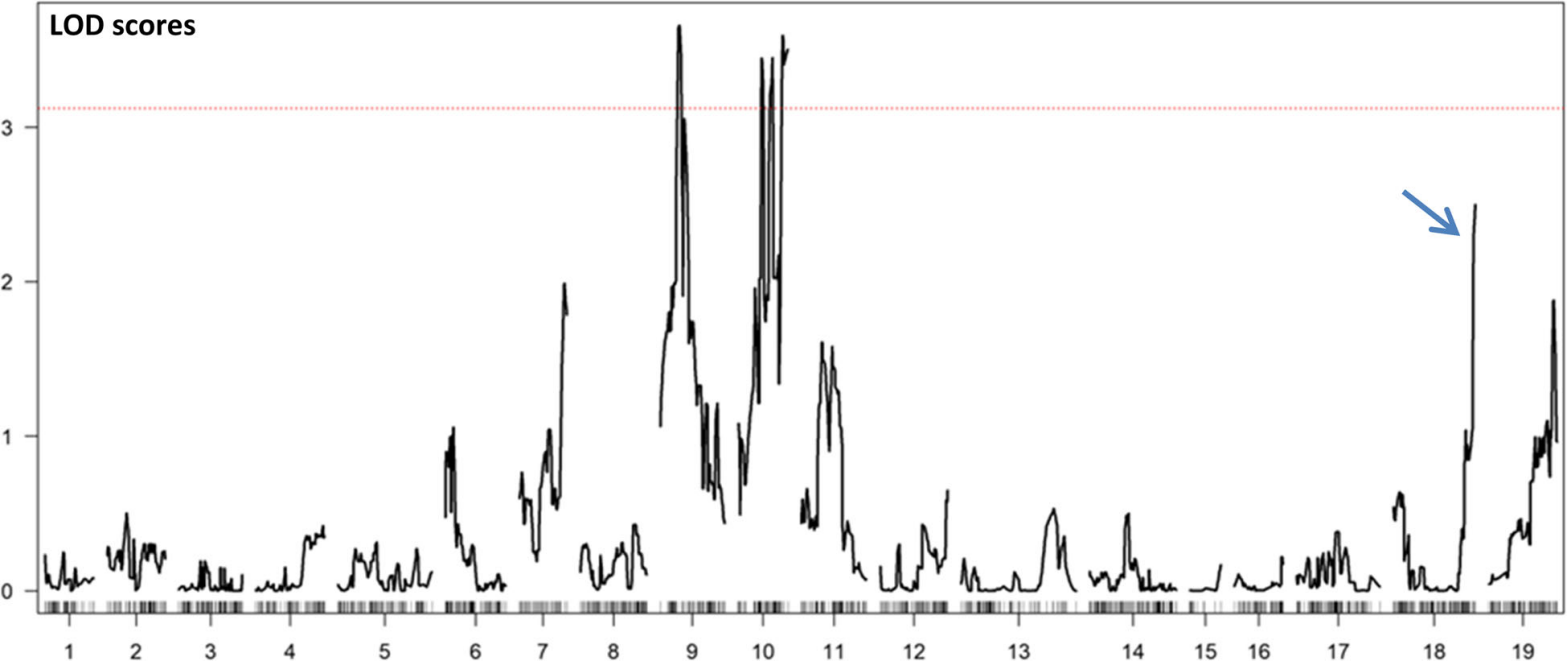
QTL analysis of the resistance to *X. index* performed on 60 BC1 individuals. The y-axis represents the LOD score obtained by the binary mapping and the x-axis represents the 19 linkage groups related to the maternal genetic map (VRH8771). Curves in plot indicate the genetic coordinate (x-axis) and LOD score (y-axis). The red dotted line represents the LOD significant threshold estimated with 1000 permutations for a level α of 0.05. The blue arrow corresponds to the genomic region below the significant threshold but with a high LOD score value

We finally performed a QTL analysis with a binary mapping model using the R/S phenotypic information from 60 BC1 individuals and the VRH8771 high-density genetic map. A first QTL explaining 22.73% of the phenotypic variance was detected on LG 9 with a LOD score of 3.66 (Table 4 – Fig. S5). VVBX-A-06, the marker identified at the QTL peak on this group, corresponds to the marker identified previously through both the BSA-type analysis (Table 4) and the proportion test (Table S4). A second QTL explaining approximatively the same proportion of phenotypic variance (21.19%) was detected on LG 10 with an equivalent LOD score (3.59) (Fig. S5). The marker SC8–03 positioned at this QTL was the same as the marker detected by the proportion test on this linkage group but it was located distantly from the three SSR markers identified in the BSA-type analysis (MRBX-27, VVIN78-SEC and VVIN85). The third QTL with the highest LOD score (13.43% of the total phenotypic variance) was located on LG 18, i.e. in the same third chromosomal location as in the BSA-type analysis and the proportion test. Marker UDV-108 identified at this QTL was also the marker obtained from both other analyses. Nevertheless, this later QTL had a LOD score of ~ 2.5 that did not reach the significant threshold value of 3.12 (Table 4). In descending order, other loci were detected on LG 7, LG 19 and LG 11 but with much lower values of LOD scores (2.0 to 1.5) (Fig. 3).

**Table 4:**
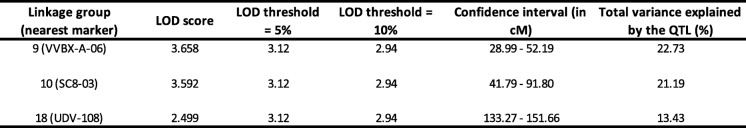
QTLs identified using a binary model in response to *X. index* performed on 60 BC1 individuals

## Discussion

### Genetic mapping of a *V. vinifera* x *M. rotundifolia* cross

*Vitis x Muscadinia* crosses have a very low fertility due to the differences in chromosome number that generate an incomplete homology between the genomes at meiosis [21]. Despite this limiting factor, a few F1 hybrids were obtained and have then been successfully backcrossed with *Vitis* species. Although the size of the progeny was limited, QTL analyses are reliable at the sight of the reproducibility of results obtained through different experimental conditions (*in planta* and in vitro) and different statistical methods (in silico BSA and QTL detection). This paper first reports the construction of high-density grapevine genetic maps for the BC1 progeny involving the *V. vinifera x M. rotundifolia* accession ‘VRH8771’ and the *V. vinifera* cv. ‘Cabernet-Sauvignon’ using both SSR and SNP types of markers. The framework maps established using SSR markers from other grapevine crosses were completed with SNP markers generated by GBS [28, 29, 34, 36, 37]. Among the whole genome and next generation sequencing techniques, GBS offers an inexpensive and robust solution for simultaneous SNP discovery and genotyping [32, 33]. In grapevine, GBS has already been used in the construction of high-resolution maps for the detection of QTLs linked to powdery and downy mildew resistance [34, 38, 39]. In the present map, the number of SNP markers was lower in the paternal parent, CS, than in the maternal parent, VRH8771. This difference is linked to the use of the *V. vinifera* CV. ‘Pinot Noir’ (PN40024) reference genome in our analyses [40, 41]. Actually, this genome is genetically closer to CS than to the *Vitis x Muscadinia* hybrid VRH8771, which explains a higher number of polymorphic markers in the maternal accession [42]. As already shown in other studies, the comparison of the marker order with their physical position in VRH8771 map confirmed the high level of macrosynteny between the *V. vinifera* and *M. rotundifolia* genomes [21, 42, 43]. The average distance between adjacent markers in all linkage groups in the maternal map was 0.98 cM, which is the same range of density reported by Teh et al. [39] and Sapkota et al. [38]. The saturation level of the maternal genetic map is close to the map established by Delame et al. [42] for a muscadine derived progeny.

### Identification of original QTLs for nodosity formation and larval development in response to root infection by *D. vitifoliae*

The response of the BC1 plants to phylloxera has been explored through both in vitro and *in planta* experiments. As expected in each experiment a wide variability of the response has been observed among replicates of a same plant individual due in particular to the vigour of the insect population. To minimize this effect, we considered the maximal values of the criteria studied as the most reliable indicators of the quantitative resistance phenotype [12]. Moreover, a linear mixed model was used to access the block effect in relation to the experimental design in randomized blocks. Thus, BLUP values were estimated and accounted for environmental effects [44]. This method has been used to optimize the statistical power for the detection of significant QTLs [45, 46]. A QTL was detected on LG 7 for nodosity and larvae numbers with an explained phenotypic variance that reached 87% in the in vitro experiment. The physiological status of the roots (which insects fed from) in each experiment, i.e. either connected to their plant (*in planta*) or completely detached from it (in vitro), was quite different. Despite this, the strongest QTLs in both experimental conditions were located on the same chromosome (LG 7). Such results are in agreement with those obtained by Bouquet [10] who reported also a good correlation of the data between the experiments conducted in vitro and in the greenhouse and/or the vineyard. Moreover, this author recorded a low ratio of phylloxera resistant plants in such *V. vinifera* x *M. rotundifolia* BC1 progenies. He suggested that this low ratio can be explained if the R factor controlling this trait is carried by a *Muscadinia* chromosome which has a low probability of pairing with its homologous *V. vinifera* chromosome in the F1 hybrids. By locating our major QTL in LG 7, the *Vitis* chromosome which had been split into the LG 7 (upper arm) and LG 20 (lower arm) in *Muscadinia*, our current data are in line with this previous hypothesis of a location of this R factor in the lower arm of chromosome 7.

Nevertheless, two other QTLs were also identified on LG 3 and LG 10 in our results, suggesting that other loci may influence or modulate nodosity formation and larval development.

The putative involvement of several QTLs in response to phylloxera has already been reported in the literature. Davidis and Olmo [8] first hypothesized that the resistance from a *M. rotundifolia* accession was controlled by more than one locus. Then Bouquet [10] suggested that grape phylloxera resistance in *M. rotundifolia* might be mediated by a semi-dominant locus regulated by three genetic modifiers. Similarly, in interspecific crosses involving different American *Vitis* species (*V. berlandieri, V. cinerea* and *V. rubra*) and *M. rotundifolia*, Boubals [7] concluded that the resistance appears to be controlled by multiple loci. Four root grape phylloxera resistance QTLs have already been identified in *Vitis* spp. In *V. cinerea*, two QTLs have been mapped in two different accessions: *RDV1* located on LG 13 in cv. ‘Arnold’ [12] and *RDV2* located on LG 14 in cv. ‘C2–50’ [5]. Two other QTLs have been detected on LG 5 and LG 10 in an F1 cross containing at least six *Vitis* species in the ancestry [13]. In our study, three new QTLs were identified from *M. rotundifolia* background. Interestingly, these seven QTLs have been mapped on six different LGs. The news QTLs were officially named *RDV6*, *RDV7*, *RDV8*, according to their position on LG7, LG3 and LG10 respectively.

### Resistance response to the dagger nematode *X. index* maps on three different LGs

The individuals were tested for their response to the dagger nematode *X. index* among successive experiments and no significant differences were found between the years of experiment, which underlines the reproducibility of the experimental protocol and the reliability of phenotyping.

Our evaluation of *X. index* resistance used a nematode reproduction factor and a root gall index assessed 10 to 12 months after inoculation. The root weight and a visual index of root development were also considered in order to study their relationships with the nematode development criteria previously mentioned. Gall index was shown to be highly correlated with the nematode reproduction factor but not with root characteristics. Such gall index rating method has been previously used in a genetic study for resistance to *X. index* in *V. arizonica*. It allowed the characterization of the early resistance conferred by the locus *XiR1* 4 to 8 weeks after inoculation in a fast greenhouse-based screening system [22, 47]. Our current data demonstrate that gall index is also a reliable criterion for rating resistance to *X. index* in longer tests (10–12 months) in order to identify R factors that should confer a more durable effect.

In our study, the distribution of the resistance levels to *X. index* was evaluated from 60 BC1 individuals. The approximate 7R:1S segregation ratio obtained suggested that three dominant and independent resistance factors might be involved in the response to the nematode. To cope with this hypothesis, a method derived from the bulked-segregant analysis [35] and performed on SSRs revealed markers on LG 9, LG 10 and LG 18. A proportion test using both SSRs and SNPs identified markers in the same three LGs and therefore confirmed previous data.

The QTL analysis performed on the same individuals supported these findings since two QTLs were identified on LG 9 and LG 10. QTL location on LG10 was not clearly estimated on this chromosome since the LOD score distribution curve showed 3 significant peaks. A third QTL was detected on LG 18 and, even though it was not significant, its LOD score value was higher than those observed on other linkage groups. These 3 QTLs were officially named *XiR2*, *XiR3* and *XiR4*, according their location on LG9, LG10, LG18 respectively. These results provide the first location of resistance factors against the vector nematode *X. index* in muscadine. On the basis of segregation patterns observed in the progenies from *Vitis* crosses involving 13 species, one-gene and two-gene modes of tolerance to *X. index* were previously reported [48], and Xu et al. [22] identified a single resistance locus, *XiR1*, on the LG 19 of *V. arizonica* using a map from 185 plants. Interestingly, some hybrids, in particular the BC1 rootstock ‘Nemadex Alain Bouquet’, derived from the *M. rotundifolia* accession ‘NC184–4’ presently studied, were shown to decrease both *X. index* numbers and GFLV infection under greenhouse and field conditions [49–52]. Consequently, we hypothesize that resistance to *X. index* conferred by this muscadine accession will be associated with a delay in GFLV transmission by the nematode to the grapevine.

### QTLs identified for response to *D. vitifoliaie* and *X. index* map to chromosomic regions enriched in resistance gene analogs

While *V. vinifera* is highly sensitive to the most critical pests and diseases for grapevine, wild *Vitis* and *Muscadinia* materials are characterized by various levels of resistance. Among all, powdery and downy mildew have been studied in details, and numerous loci controlling partial or total resistance to both diseases have been identified [53]. Interestingly, some of the chromosomal regions identified in our study as carrying R factors to *D. vitifoliae* and *X. index* already encompass QTLs identified in response to other major grapevine diseases. LG 18 contains many loci detected for resistance to downy mildew *Plasmopara viticola* and powdery mildew *Erysiphe necator* in mucadine [54, 55] or *Vitis* spp. [38, 56–58]. Thus, among the markers flanking the *Rpv3* (resistance to *P. viticola)* locus identified from ‘Regent’ genotype by van Heerden and collaborators [59] on LG 18, is the UDV-108 marker which has been identified in our study with the maximum LOD score in one of the three QTLs highlighted in response to *X. index*. Additionally, in *Vitis* spp., the loci *rpv7* (resistance to downy mildew) and *rpv5* and *Ren6* (downy and powdery mildew) have been reported on LG 7 and LG9 respectively [56, 60, 61]. These three chromosomes have been previously demonstrated to be enriched in NBS-LRR genes [62]. The current development of sequencing technologies should contribute to the comparison of *Vitis* genomes and to gather more precise knowledge about these resistance genes in order to use them in breeding new cultivars with multi-pest resistances.

## Conclusions

With the objective of grapevine rootstock breeding, our data open the way to the use of muscadine as a source for the obligate phylloxera R feature and as an R source to *X. index* in order to delay GFLV transmission to grapevine by this nematode. In this work, we could successfully identify root resistances against phylloxera and *X. index* and map the first cognate QTLs from the *M. rotundifolia* source ‘NC184–4’. Thus QTLs were identified on LG 7 completed by LG 3 and LG 10 in response to phylloxera and on LG 9, LG 10 and LG 18 in response to *X. index*. These newly identified trait-linked loci should now be tested in alternative crosses with *M. rotundifolia*. In the future, validated markers at those loci may allow their use in a marker-assisted breeding approach for producing new rootstocks with durable resistances.

## Methods

### Plant material

We used the backcross 1 (BC1, population Bdx0227) segregating population of 135 individuals (Table S1) from the cross between the hybrid VRH8771 [(‘Cabernet-Sauvignon’ *x* ‘Alicante Bouschet’) *x M. rotundifolia* cv. *NC184–4*] and *V. vinifera* cv. ‘Cabernet-Sauvignon’ (VRH8771 x CS). This cross has been initiated by Alain Bouquet at INRAE Montpellier since 2005 and continued at INRAE UMR EGFV (Bordeaux, France) after 2008. The female parent VRH8771 was initially obtained from a cross made in 1975–1976 at INRAE Bordeaux with the intra-*vinifera* hybrid previously obtained in Bordeaux as the mother (accession number 8606), and pollen from Muscadine genotype NC184–4 obtained from Nesbitt W.B. (Raleigh, University of North Carolina, USA) [63]. VRH8771 is resistant to *D. vitifoliae* and *X. index* whereas *V. vinifera* ‘Cabernet-Sauvignon’ (CS), the male parent, is susceptible [49]. Parental genotypes and BC1 individuals are maintained at the INRAE germplasm repository (Bordeaux, France).

### Insect and nematode material

For *D. vitifoliae*, all experiments were conducted with the isofemale clone ‘Pcf7’. The original population was sampled in 2010 in a commercial vineyard at Pineuilh (Gironde, France) on *V. vinifera* cv. ‘Cabernet franc’ scions grafted on SO4 rootstock (*V. berlandieri x V. riparia*) and maintained at INRAE UMR SAVE (Bordeaux, France) on leaves of the American variety ‘Harmony’, a complex hybrid between Dog-Ridge (*V. champinii)* and ‘1613C’ (*V. labrusca x V. riparia x V. vinifera*) and on root pieces of *V. vinifera* cv. ‘Cabernet Sauvignon’, both genotypes originating from INRAE germplasm repository, multiplied as cuttings and grown under greenhouse conditions.

For *X. index*, all experiments were conducted with the isofemale line ‘Frejus’. The original population has been sampled in a GFLV-infected grapevine field in Frejus (Provence, France). Using the population grown on grapevine in the greenhouse, the line had been created from a single female inoculated on a fig plant previously grown from in vitro.

### Experimental designs and phenotyping

#### Phylloxera assays

An *in planta* assay was performed with 89 BC1 individuals. Two control genotypes were also tested: *V. vinifera* cv. ‘Pinot Noir’ and the rootstock ‘Börner’ (*V. riparia x V. cinerea*) as susceptible and resistant genotypes respectively. This experiment was organized according to a randomized complete block design with three experimental blocks, each block being an independent randomization of one replicate of each BC1 individual. Plants were grown in individual pots of 1 L in a soil substrate composed of at least 50% clay with pebbles at the bottom to improve drainage. The soil was sterilized by autoclaving in order to prevent cross contamination with other phylloxera population sources. Each pot was covered with an insect-proof transparent plastic bell and an automatic watering system was adapted (Fig. S6 A). One hundred phylloxera eggs of ‘Pcf7’ clone, previously grown on root pieces of *V. vinifera* cv. ‘Cabernet Sauvignon’ for two generations, were deposited on a moistened and sterilized filter paper near the root (~ 3 cm depth) of each pot. Three months after inoculation, the plants were uprooted and the nodosity number was counted (Fig. S6 B). The maximal number of nodosities scored among the three replicates of each BC1 individual was considered as the most reliable indicator of the quantitative resistance phenotype.

An in vitro assay was also performed according to Pouget [64] to assess the larval development of phylloxera on 37 BC1 individuals. Five woody root pieces per individual, 6 to 7 cm long, were arranged in small bundles (a contact between all the roots is required) on a disk of dampened blotting paper in a Petri dish sealed with parafilm. Three replicates were realized for each individual. A fungicide treatment (Ridomil Gold, Syngenta® - 2.3 g/L) was carried out on the roots to prevent *Botrytis cinerea* infection. In each Petri dish, 50 phylloxera eggs were deposited on the roots pieces. Then Petri dishes were incubated at 25 °C in the dark. The number of larvae that have developed on the five root pieces was counted 1 month after inoculation. The maximal number of larvae scored among the three replicates was considered as the best indicator of the quantitative resistance phenotype [12].

#### X. index assays

A total of 60 BC1 individuals were evaluated during five successive experiments conducted between 2010 and 2017 (Table S4). Three reference genotypes were also tested: the susceptible *V. rupestris* cv. ‘du Lot’, the resistant BC1 rootstock ‘Nemadex Alain Bouquet’ from the cross ‘VRH8773 (*V. vinifera x M. rotundifolia*) x 140 Ru (*V. berlandieri x V. rupestris*)’ and the susceptible to intermediate genotype ‘VRH8624’. VRH8773 is a brother clone of VRH8771, and VRH8624 in an F1 hybrid (*V. vinifera x M. rotundifolia*) whose muscadine parent is the accession ‘Trayshed’ [65].

For each evaluation, homogenous hardwood cuttings of the individuals were rooted annually in alveolated plates in the nursery at INRAE UMR EGFV (Bordeaux, France) in February. In May, plants were delivered to INRAE UMR ISA (Sophia-Antipolis, France), planted individually into 2-L pots with six replicates per individual and grown in a greenhouse. At the end of June, each pot was inoculated with a fixed number of nematodes that ranged from 300 to 900 depending on the year of experiment (Table S5). The plants were grown for ten to twelve months, which is the time that allows approximately three to four nematode developmental cycles over two successive calendar years.

At harvest, the aerial part of the plant was cut at the collar level and removed and each pot was hermitically placed into a plastic bag and stored in a cold chamber at 6 °C. This stopped plant and nematode development simultaneously in all individual replicates until plant and nematode ratings. Ratings were done sequentially, i.e. replicate after replicate. Total soil of each 2-L pot was recovered in a 10-L bucket, over which plant roots were washed individually with caution under tap water. The entire root system of each plant was rated for its root development (RD) based on a 0–5 scale and its fresh root weight (RW) was also measured. Root galling was rated for each plant using a 0–5 gall index (GI) scale derived from studies for resistance to the root-knot nematode *Meloidogyne* spp.: 0 = no gall; 1 = 1–10%; 2 = 11–30%; 3 = 31–70%; 4 = 71–90%; 5 > 90% of root system galled [66]. Nematodes of each plant were extracted from the total soil suspended in the bucket using an adapted Oostenbrink method [67]. In the two first experiments (2010–2011 and 2011–2012), final nematode numbers were counted under a binocular microscope. Then the ratio between nematode final and initial numbers was calculated to evaluate the mean nematode reproduction factor (RF) for each BC1 individual and the parental and reference genotypes. Individuals were classified as resistant (R) when their RF value was lower than 1 and susceptible (S) when their RF value was equal or above 1. As the two first experiments showed that RF ratings were significantly correlated with GI ratings (see Results section), no nematode extraction was performed for the three last experiments (2012–2013, 2015–2016 and 2016–2017) and individuals were classified directly as R or S from their visual symptoms.

### Treatment of phenotypic data sets

#### Phylloxera assays

The number of nodosities and the number of larvae were explored using the following generalized mixed model:

$$ {\mathrm{P}}_{\mathrm{ij}}=\upmu +\mathrm{individual}+{\mathrm{block}}_{\mathrm{j}}+{\upvarepsilon}_{\mathrm{ij}} $$

where *P*_*ij*_ is the observed phenotype, *μ* is the overall mean of the phenotypic data, ‘individual’ corresponds to the genetic differences among the BC1 individuals, ‘block’ accounts for the differences in microenvironmental conditions among the three blocks, and *ε*_*ij*_ is the residual term (R _LME4_ package). The factor ‘individual’ was treated as a random factor, whereas the factor ‘block’ was treated as a fixed factor. The ‘Best linear unbiased predictions’ (BLUP) of random effects were extracted from the selected generalized mixed model. The BLUP values were noted by the name of the trait preceded by the word BLUP.

For each quantitative phenotypic trait, broad sense heritability was estimated using the formula *h*^*2*^ *= σ*^*2*^_*g*_*/[σ*^*2*^_*g*_ *+ σ*^*2*^_*e/n*_*]*, where *σ*^*2*^_*g*_ is the genetic variance, *σ*^*2*^_*e*_ is the environmental variance and *n* is the number of plants per accession.

#### Nematode assays

All principal component analyses (PCA) and parametric and non-parametric statistical tests were performed using R version 3.4.0. Statistical significance was set at *P* < 0.05.

### Mapping of the resistance traits

#### Simple sequence repeats (SSR) markers

DNA was extracted from 50 to 100 mg of young leaves grown in a greenhouse from 90 BC1 individuals (Table S1). Leaves were ground in 5 mL of a first buffer extraction containing sodium metabisulfite [sodium metabisulfite 20 mM, Tris-HCl pH 8 0.2 M, EDTA pH 8 70 mM, NaCl 2 M]. 500 μL of this homogenate was incubated with 450 μL of a second buffer with CTAB [CTAB 2%, NaCl 1.4 M, EDTA pH 8 20 mM, Tris-HCl pH 8 0.1 M] during 1 h at 65 °C. Then the solution was centrifuged for 30 min at 13,000 rpm at 4 °C. 500 μL of supernatant was sampled and cleaned with the same volume of a solvent chloroforme:octanol (24:1). After 20 min of spin at 13,000 rpm at 4 °C, we recovered 300 μL of the aqueous phase in 450 μL of a solution of isopropanol: ammonium acetate (2:1). We left 1 h at 4 °C and then we centrifuged at 13,000 rpm for 20 min at 4 °C. We discarded the solution and washed the pellet with 70% ethanol v/v (two times). After centrifugation (13,000 rpm for 20 min at 4 °C), we discarded the ethanol, and precipitated the pellet in 100 to 200 μL of TE 0.1 X [Tris HCl 10 mM, EDTA 1 mM]. DNAs were quantified by Nanodrop™.

A total of 217 primers pairs were used: 61 VMC (Vitis Microsatellite Consortium, managed through AGROGENE, Moissy Cramayel, France), 7 VVMD [68], 1 VVS [69], 6 SC08 [70], 1 VrZAG [71], 7 VVC [69], 59 VVI [72], 11 MRBX [73], 21 UDV [74] and marker Gf13–9 [12]. Two new series of SSRs, VVBX and VVBX-A, were designed from the genome 12X of *V. vinifera* cv. ‘Pinot Noir’ (PN40024) using Primer3 software [75]. Primers characteristics of VVBX and VVBX-A markers are reported in Table S6.

PCRs were performed by a single reaction with M13-tailed forward primer [76] conjugated with four different dyes (6FAM™, VIC®, NED™ and PET®) in 15 μl reaction volume containing: 5 ng of DNA template, 1.5 μL of 10xPCR reaction buffer, 2 mM of MgCl2, 0.2 mM of each dNTP, 0.05 μM of M13 tailed SSR forward primer, 0.2 μM of reverse primer, 0.2 μM of dye conjugated with M13 primer, and 0.2 U of JumpStart™ Taq DNA Polymerase (Sigma-Aldrich). Amplification conditions were as follows: 5 min initial denaturation step at 95 °C followed by 2 cycles (30 s denaturation at 95 °C, 1.5 min annealing at 60 °C or 56 °C and 1 min extension at 72 °C) followed by 35 cycles (30 s denaturation at 95 °C, 30 s annealing at 60 °C or 56 °C and 1 min extension at 72 °C) then followed by 10 min final extension at 72 °C. Visualization was performed by a 3730 DNA analyzer (Applied Biosystem™). Eight to sixteen PCR products were pooled, according to the size of SSRs and the dyes, and analyzed in a single run. Electropherograms were analyzed using free software STRand. Any ambiguous genotypes were re-run, re-amplified or left as unknown.

#### Single nucleotide polymorphisms (SNP) markers

Two foliar disks (1.5 cm of diameter) of 128 BC1 individuals grown in a greenhouse were sampled (Table S1). DNA extractions were realized on dried leaf tissues using the same protocol as described by Cormier et al. [77]. Genotyping-by-sequencing (GBS) was performed as described by Elshire et al. [32] (Keygene N.V. owns patents and patent applications protecting its Sequence Based Genotyping technologies) integrating two 96-well plates across 96 barcodes for library preparation. The genomic library was prepared using ApeKI restriction enzyme. Paired-end sequencing of 150 bp reads was performed on an Illumina HiSeq3000 system (at the GeT-PlaGe platform in Toulouse, France).

Raw reads were checked with FastQC [78], demultiplexed with a custom script (https://github.com/timflutre) and cleaned with CutAdapt [79]. Cleaned reads were then mapped to the *V. vinifera* cv ‘Pinot Noir’ (PN40024) genome assemblies for SNP calling. Alignment on this genome was performed using Burrows-Wheeler Aligner maximal exact match (BWA-MEM) with default parameters [80], SAMtools and Picard (http://broadinstitute.github.io/picard/). SNP calling was performed with GATK using the hardfilter parameters [81–83].

VCFtools was used to remove SNPs with a quality score < 200 and with depth values < 10 [84]. Quality-filtered SNPs were analyzed with the *major_minor* and *get_pseudo_test_cross* scripts from HetMappS pipeline to identify pseudo-testcross markers [85]. In the variant call format (VCF) output file only sites with less than 10% missing data were retained. Individuals with more than 50% missing data and those with genotype frequencies different from expected 1:1 marker segregation were discarded. Two sets of markers were obtained corresponding to the two parental genotypes.

#### Map construction

Individual maps were constructed for each parental genotype following a double pseudo-testcross strategy [86]. Marker segregation was analysed with regard to goodness-of-fit to the expected Mendelian ratio using the Chi-square test (*P* < 0.05). Marker types of *lm x ll* and *nn x np* were retained for construction of maternal and paternal maps, respectively. Genetic maps and marker order were determined using the maximum likelihood (ML) algorithm with Haldane function and default parameters of JoinMap®4.1 software [87, 88]. Linkage groups (LGs) were constructed with a minimum threshold logarithm of odds (LOD) score of 6.0. LGs were grouped and numbered based on their corresponding physical chromosome numbers [41].

### Methods for resistance mapping

#### Phylloxera assays

The maximum number of nodosities (*in planta* assay), the maximum number of larvae (in vitro assay) and the BLUP values associated were used as the quantitative scores of susceptibility/resistance response.

Detection of quantitative trait loci (QTLs) was performed with the one-dimension scan function, *scanone*, of R/qtl software using a normal model but also a non-parametric analysis and the expectation-maximization (EM) algorithm method depending on the normality of the data [88, 89]. Multipoint genotype probabilities were calculated beforehand using *calc.genoprob* with step = 1 and default parameters. Logarithm of odd score (LOD) significance threshold was estimated with 1000 permutations and for a significant level α of 0.05. An interval estimate of the location of each QTL was calculated using the 1.5-LOD intervals method of Rqtl [90]. The percentage of the phenotypic variation explained by a QTL corresponds to the regression value R^2^ taken at its peak LOD score.

#### Nematode assays

Based on SSR markers, an in silico method derived from the Bulked Segregant Analysis (BSA) was performed [35]. In the VRH8771 x CS progeny, polymorphic markers were distributed into either 2 (ab), 3 (abc) or 4 (abcd) allelic forms. From each of the markers screened, markers retained were those for which an allele was detected in the resistant parent (VRH8771) and in part of the resistant BC1 individuals but in none of all susceptible BC1 individuals.

The resistant and susceptible phenotypes were converted to values equal to 0 and 1, respectively. The one-dimension scan function, *scanone*, of R/qtl software was performed with the argument *model = ‘binary’* [90]*.* LOD significance threshold, the QTL interval and the R^2^ value were obtained with the procedure described in the previous paragraph. A number of 47 phenotyped individuals were used for the BSA analysis with SSR markers whereas the 60 total individuals phenotyped were used for QTL analysis with SSR and SNP markers.

## Supplementary information

**Additional file 1: Table S1.** Characteristics of the 135 BC1 individuals. **Table S2.** Statistical analyzes comparing data between 2010 and 2011 and 2011–2012 experiments. **Table S3.** Spearman correlations of the four criteria (RD – RW – RF – GI) in response to *X. index.***Table S4.** Results of the proportion test performed on 60 BC1 individuals in response to *X. index.***Table S5.** Characteristics of the experiments in response to *X. index* carried out over five independent years. **Table S6.** Primers characteristics.

**Additional file 2: Figure S1.** Maternal genetic map related to VRH8771. **Figure S2.** Paternal genetic map related to CS. **Figure S3.** Variation of genetic distance depending on physical distance on the VRH8771 (A) and CS (B) genetic maps. **Figure S4.** Principal component analysis (PCA) of the 35 F1 individuals tested in 2010–2011 and 2011–2012 experiments with root system development (RD), root weight (RW), nematode reproduction factor (RF) and gall index (GI). **Figure S5.** QTL analysis of the resistance to *X. index* performed on 60 BC1 individuals. The results of the analysis performed on LG 9 and LG 10 are presented. The y-axis represents the LOD score obtained by the binary mapping and the x-axis represents the 19 linkage groups related to the maternal genetic map (VRH8771). Curves in plot indicate the genetic coordinate (x-axis) and LOD score (y-axis). The red dotted line represents the LOD significant threshold estimated with 1000 permutations for a level α of 0.05. **Figure S6.***In planta* experiment (A) illustration of the experimental device with each plant grown in an individual pot covered by a transparent plastic bell and (B) example of nodosities developed on roots (red arrows).

## Data Availability

The data sets during the current study are available from the corresponding author.

## Abbreviations

BLUP: Best linear unbiased predictions
BSA: Bulked segregant analysis
EM: Expectation-maximization
GBS: Genotyping-by-sequencing
LG: Linkage group
LOD: Logarithm of odds ratio
ML: maximum likelihood
NGS: Next-generation sequencing
PCA: Principal component analysis
QTL: Quantitative trait locus
SNP: Single nucleotide polymorphism
VCF: Variant call format
