# Supplementary material for: Characterization of genetic determinants of the resistance to phylloxera, Daktulosphaira vitifoliae, and the dagger nematode Xiphinema index from muscadine background
Source: BMC Plant Biol. 2020 May 12;20:213. doi: 10.1186/s12870-020-2310-0 (PMC7218577; doi:10.1186/s12870-020-2310-0)
Supplement: Supplementary file 1 — Additional file 1: Table S1. Characteristics of the 135 BC1 individuals. Table S2. Statistical analyzes comparing data between 2010 and 2011 and 2011–2012 experiments. Table S3. Spearman correlations of the four criteria (RD – RW – RF – GI) in response to X. index.Table S4. Results of the proportion test performed on 60 BC1 individuals in response to X. index.Table S5. Characteristics of the experiments in response to X. index carried out over five independent years. Table S6. Primers characteristics. [file 12870_2020_2310_MOESM1_ESM.docx]

**Table S1 Characteristics of the 135 BC1 individuals**

**Table S2 Statistical analyzes comparing data between 2010-2011 and 2011-2012 experiments**

The non-parametric Wilcoxon signed-rank test was performed on four traits: root system development (RD), weight of the roots (RW), nematode reproduction factor (RF) and gall index (GI)

Signif.codes: 0 ‘***’ 0.001 ‘**’ 0.01 ‘*’ 0.05 ‘.’ 0.1 - *NS*, not significant


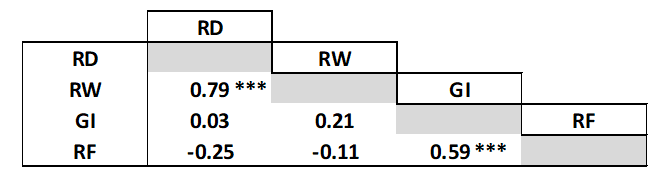


**Table S3 Spearman correlations of the four criteria (RD – RW – RF – GI) in response to *X. index***

Signif.codes: 0 ‘***’ 0.001 ‘**’ 0.01 ‘*’ 0.05 ‘.’ 0.1

**Table S4 Results of the proportion test performed on 60 BC1 individuals in response to *X. index***

The results presented are related to the markers for which the p-values are statistically significant at α = 5%

**Table S5 Characteristics of the experiments in response to *X. index* carried out over five independent years**

RF: reproduction factor, GI: gall index, RW: root weight, RD: root development, R/S: resistance *vs.* susceptible qualitative phenotyping

**Table S6 Primers characteristics**
