# Supplementary material for: Characterization of genetic determinants of the resistance to phylloxera, Daktulosphaira vitifoliae, and the dagger nematode Xiphinema index from muscadine background
Source: BMC Plant Biol. 2020 May 12;20:213. doi: 10.1186/s12870-020-2310-0 (PMC7218577; doi:10.1186/s12870-020-2310-0)
Supplement: Supplementary file 2 — Additional file 2: Figure S1. Maternal genetic map related to VRH8771. Figure S2. Paternal genetic map related to CS. Figure S3. Variation of genetic distance depending on physical distance on the VRH8771 (A) and CS (B) genetic maps. Figure S4. Principal component analysis (PCA) of the 35 F1 individuals tested in 2010–2011 and 2011–2012 experiments with root system development (RD), root weight (RW), nematode reproduction factor (RF) and gall index (GI). Figure S5. QTL analysis of the resistance to X. index performed on 60 BC1 individuals. The results of the analysis performed on LG 9 and LG 10 are presented. The y-axis represents the LOD score obtained by the binary mapping and the x-axis represents the 19 linkage groups related to the maternal genetic map (VRH8771). Curves in plot indicate the genetic coordinate (x-axis) and LOD score (y-axis). The red dotted line represents the LOD significant threshold estimated with 1000 permutations for a level α of 0.05. Figure S6.In planta experiment (A) illustration of the experimental device with each plant grown in an individual pot covered by a transparent plastic bell and (B) example of nodosities developed on roots (red arrows). [file 12870_2020_2310_MOESM2_ESM.docx]

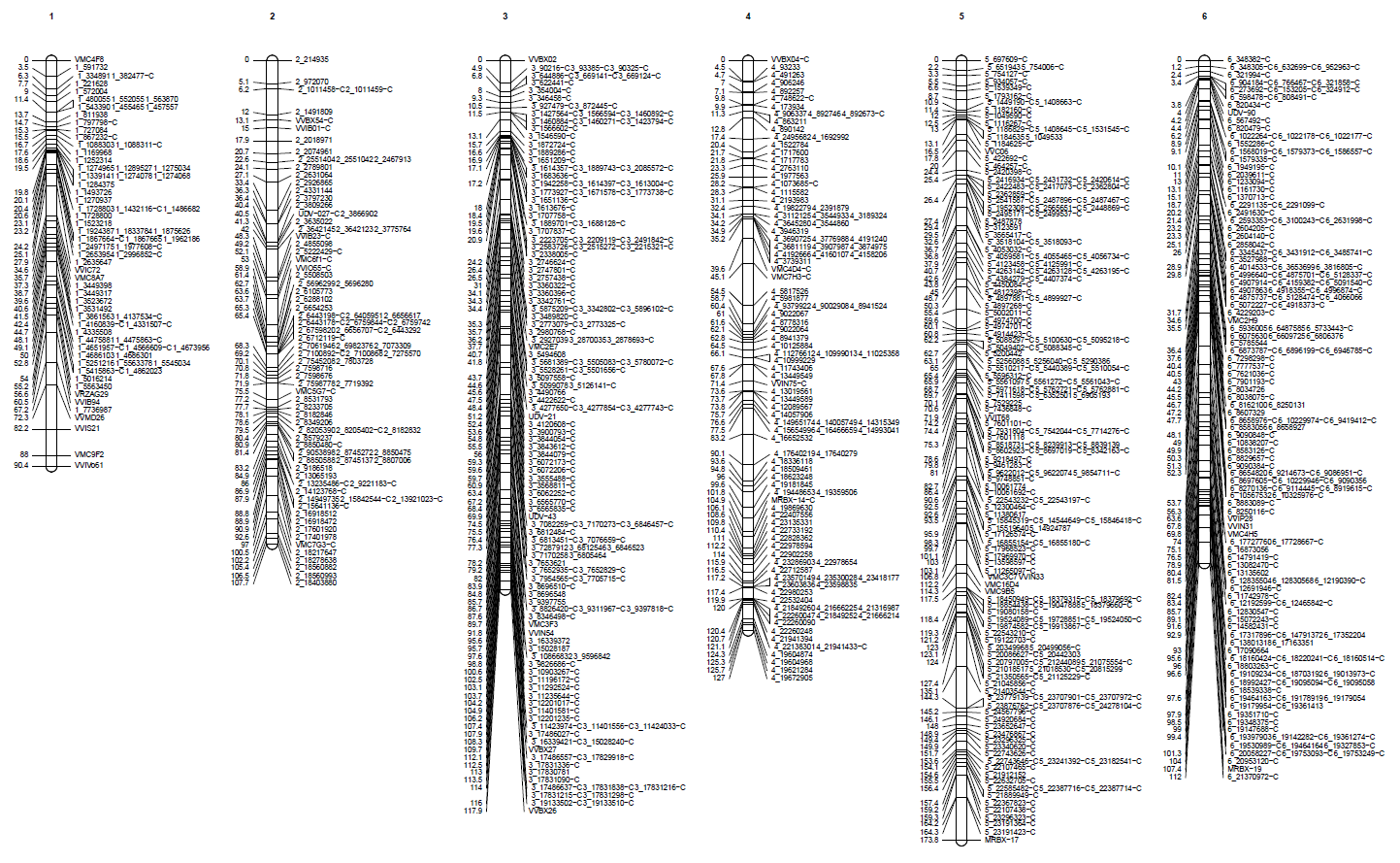

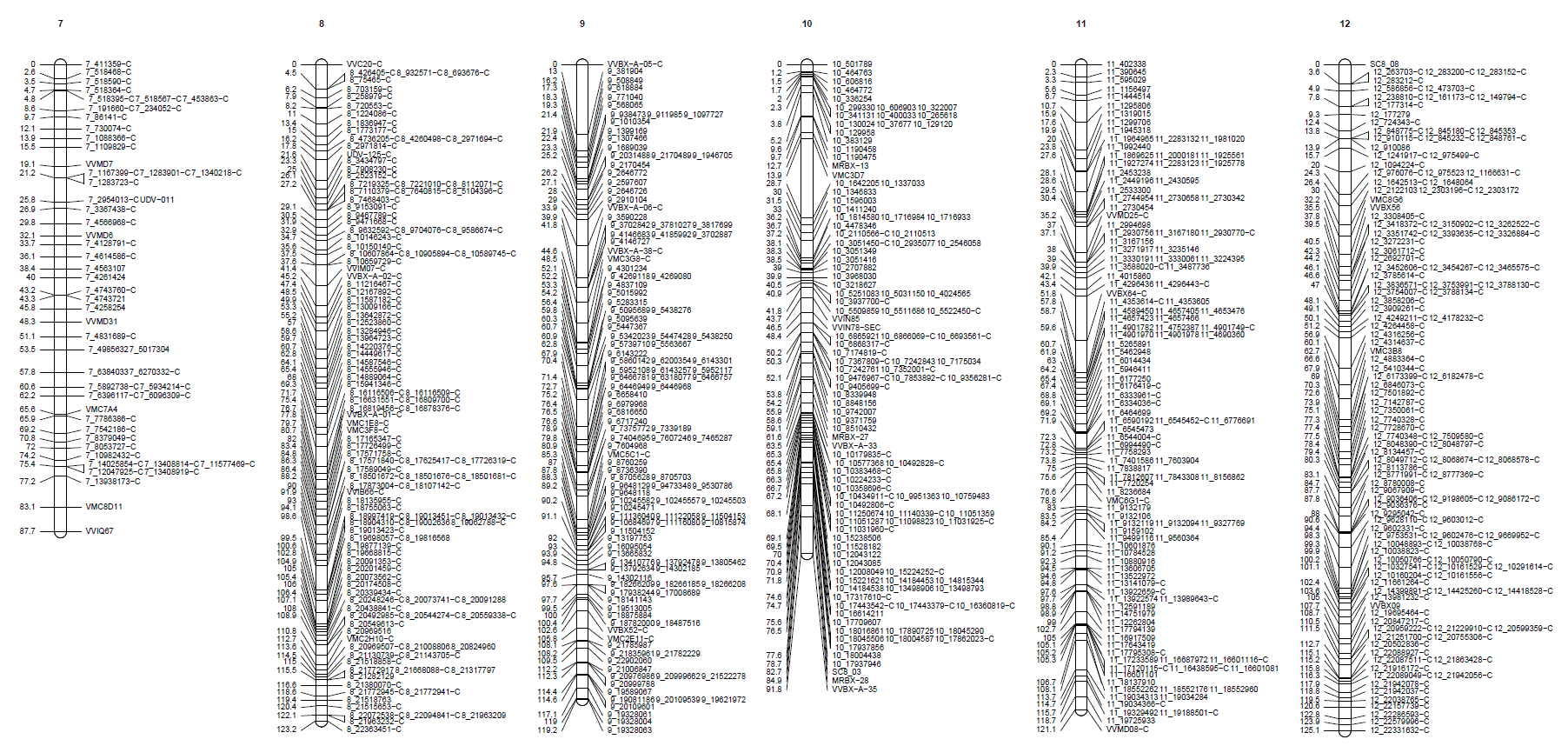


**1**

**2**

**3**

**4**

**5**

**6**

**7**

**8**

**9**

**10**

**11**

**12**


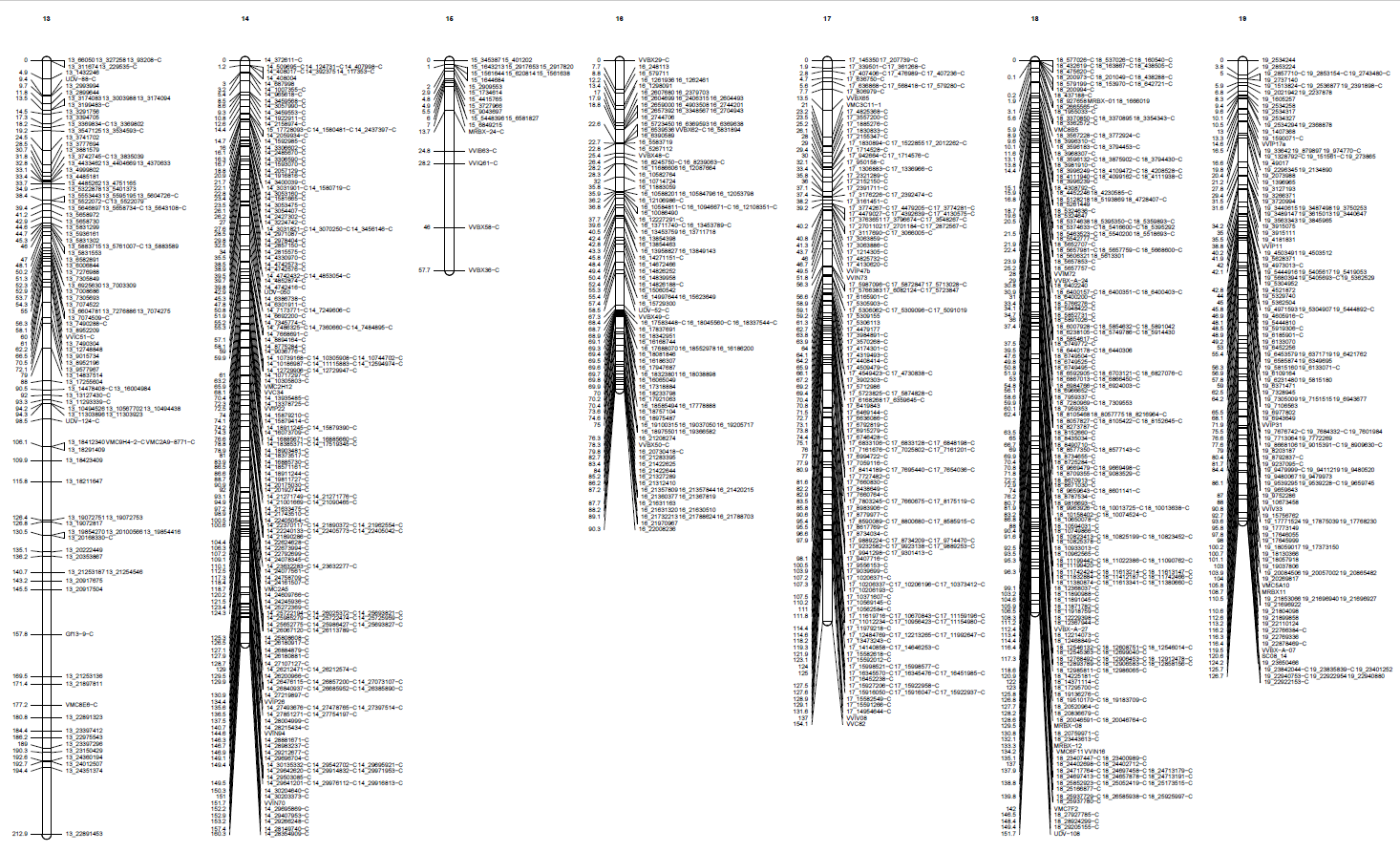


**13**

**14**

**15**

**16**

**17**

**18**

**19**

**Fig. S1 Maternal genetic map related to VRH8771**

Each linkage group is represented by a bar with the genetic positions on the left side and marker names on the right side


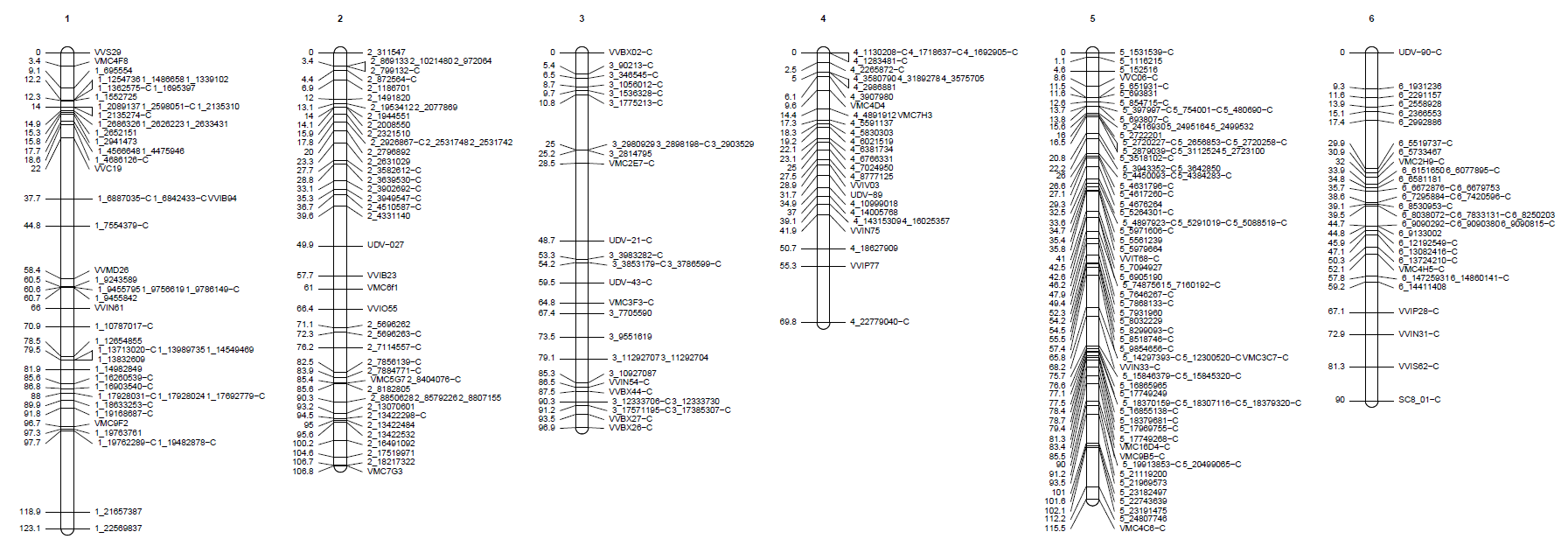


**1**

**2**

**3**

**4**

**5**

**6**


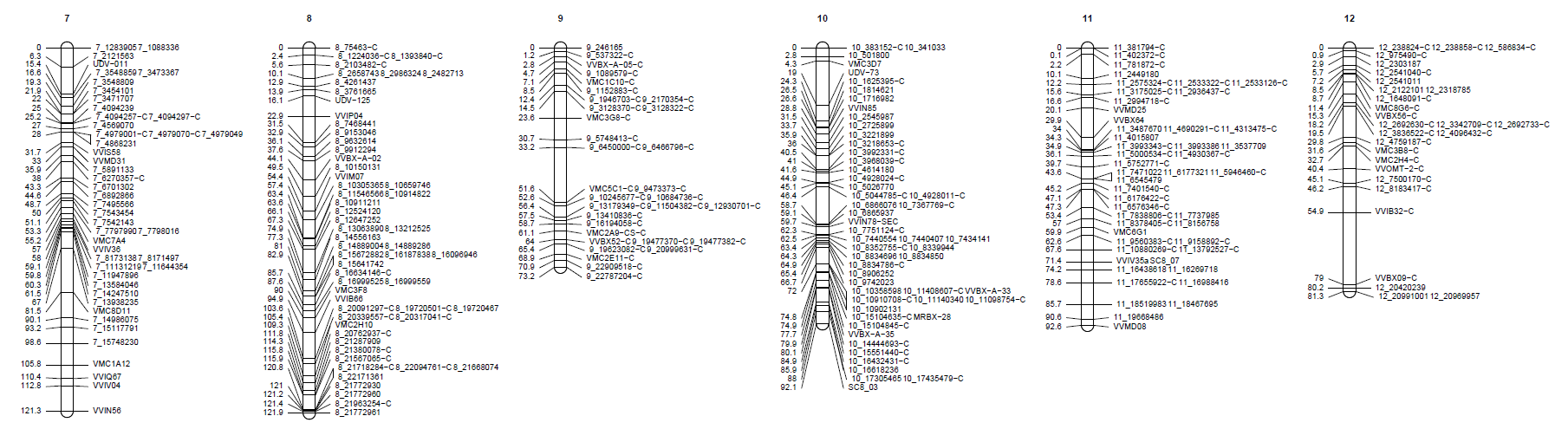


**7**

**8**

**9**

**10**

**11**

**12**


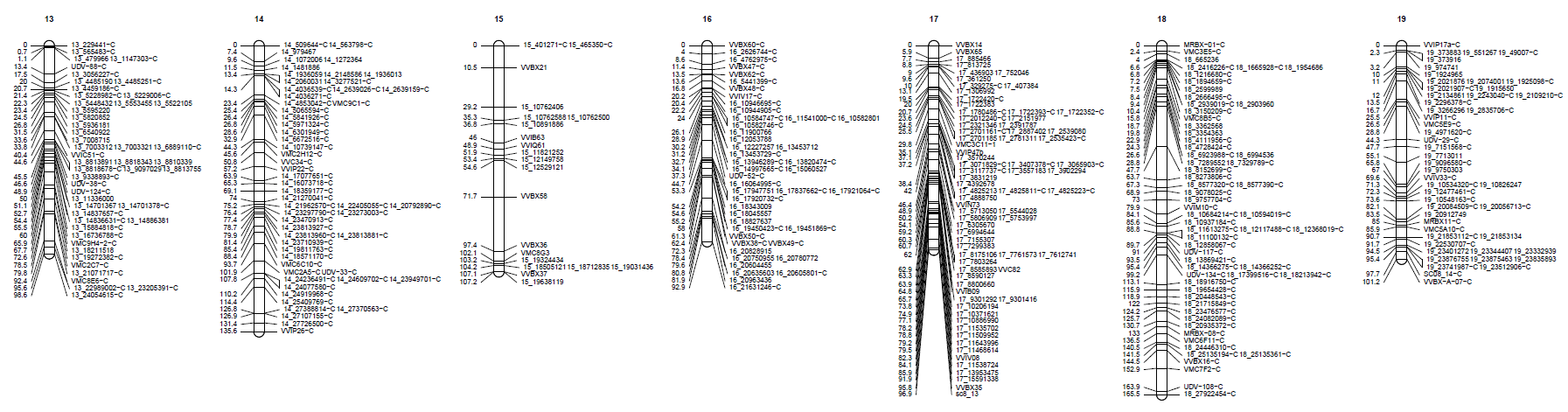


**13**

**14**

**15**

**16**

**17**

**18**

**19**

**Fig. S2 Paternal genetic map related to CS**

Each linkage group is represented by a bar with the genetic positions on the left side and marker names on the right side.


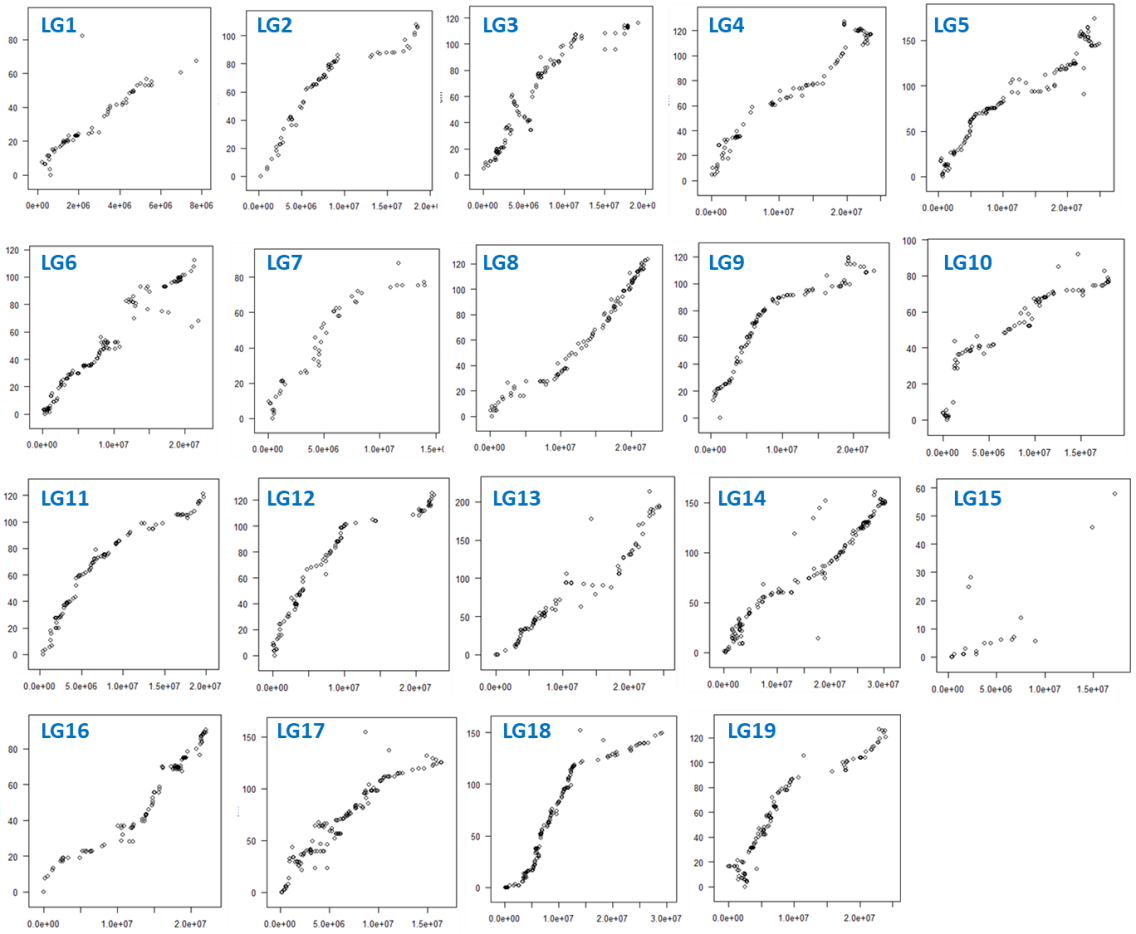


**A**

**Fig. S3 Variation of genetic distance depending on physical distance on the VRH8771 (A) and CS (B) genetic maps**

The y axis represents the genetic distances (in cM) and the x axis represents the physical distances (in bp) related to the *V. vinifera* cv ‘Pinot Noir’ (PN40024). Each plot represents an individual linkage group


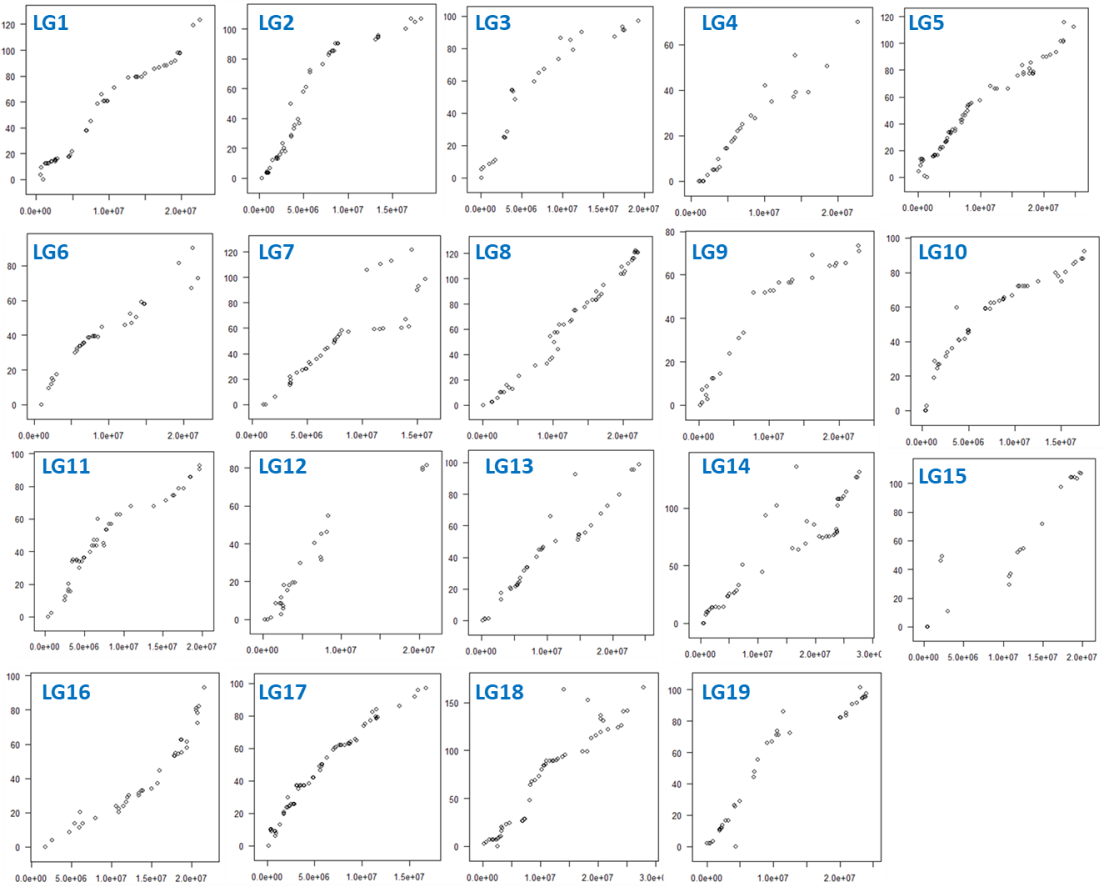


**B**


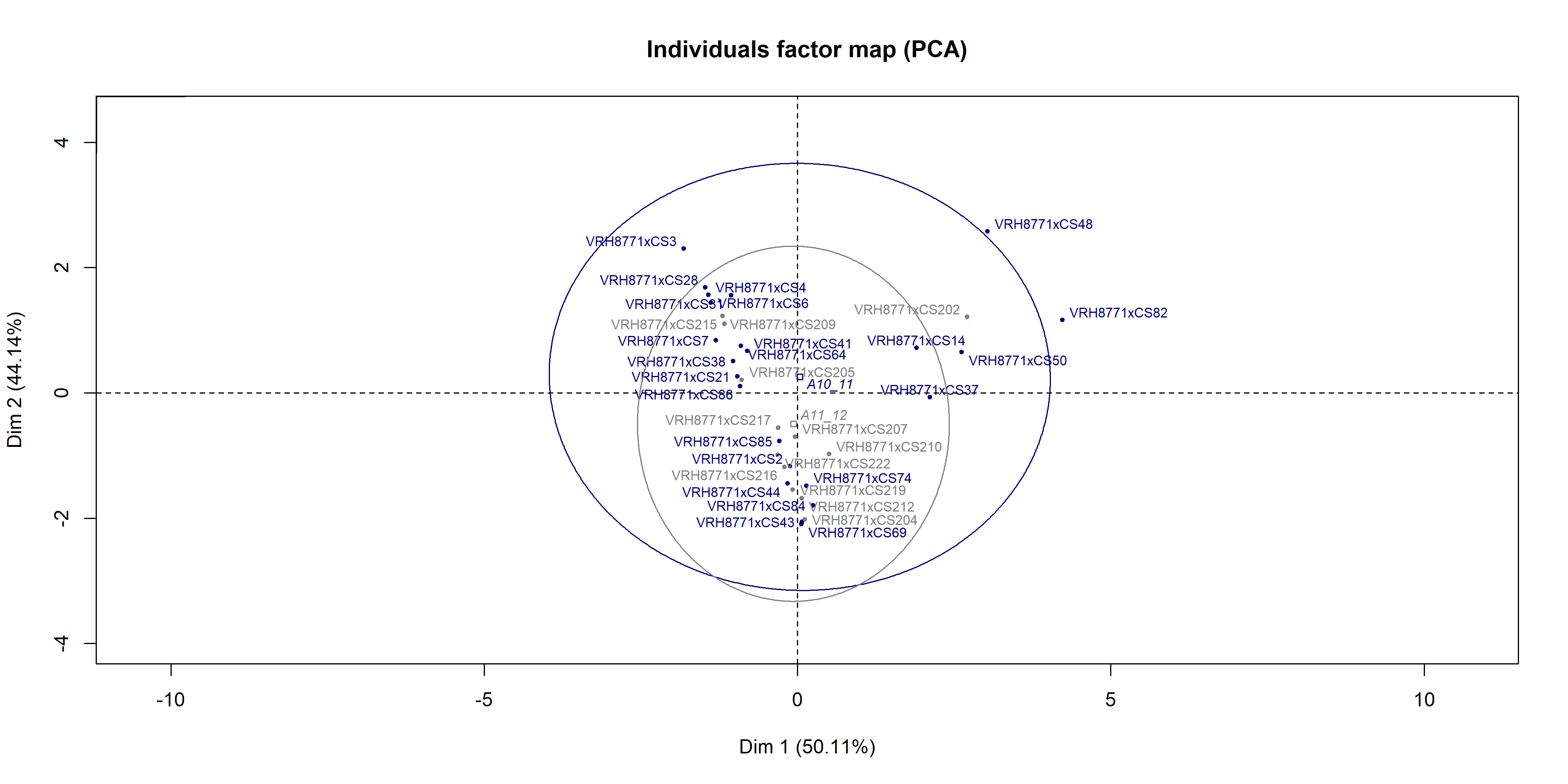


**Fig. S4 Principal component analysis (PCA) of the 35 F1 individuals tested in 2010-2011 and 2011-2012 experiments with root system development (RD), root weight (RW), nematode reproduction factor (RF) and gall index (GI).**

The two major principal components that accounted for 94.25 % of the variance have been plotted. The individual factor map is represented: the BC1 individuals were assigned to two groups according to the year of experiment: 2010-2011 in blue and 2011-2012 in gray.


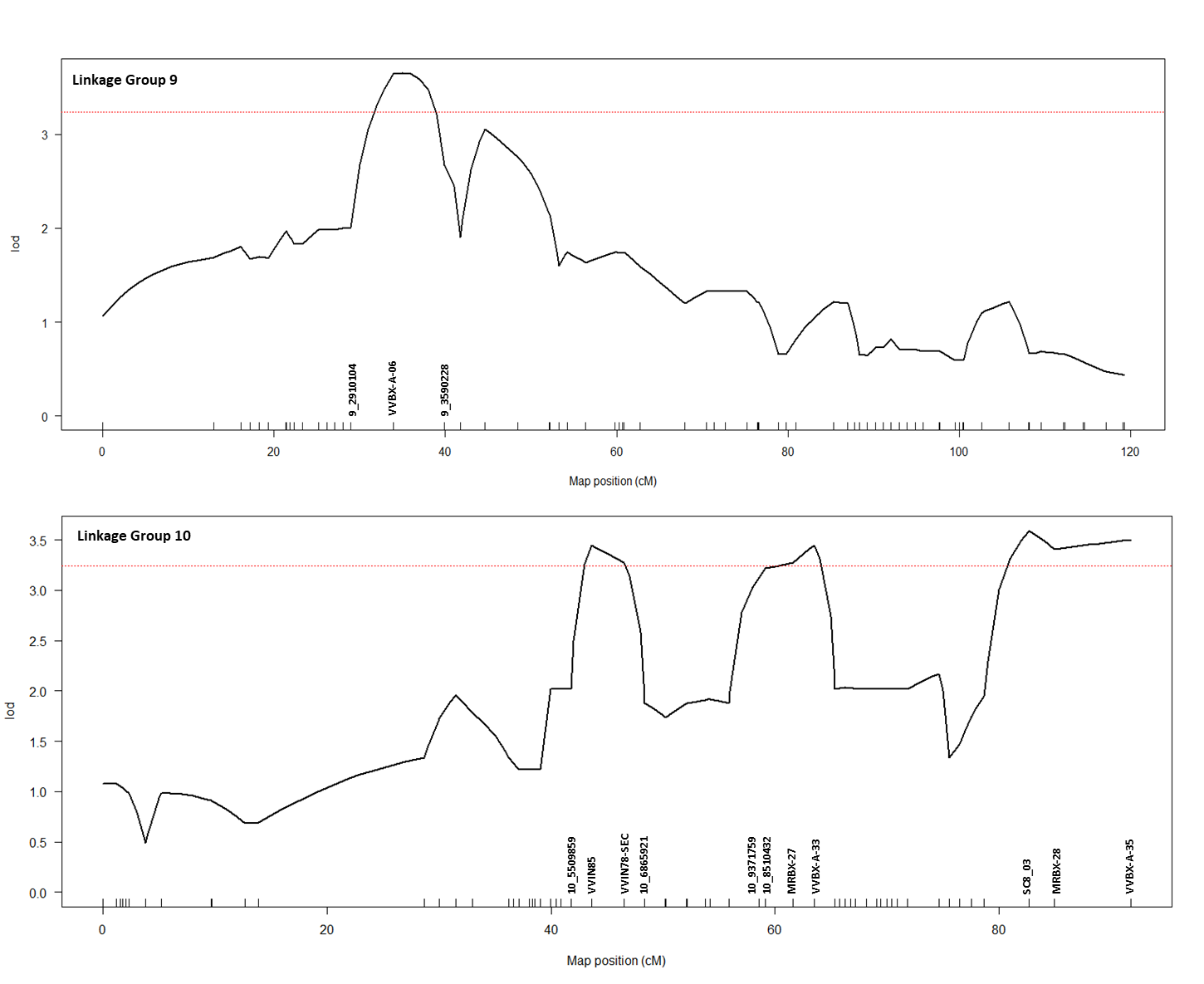


**Fig. S5 QTL analysis of the resistance to *X. index* performed on 60 BC1 individuals. The results of the analysis performed on LG 9 and LG 10 are presented. The y-axis represents the LOD score obtained by the binary mapping and the x-axis represents the 19 linkage groups related to the maternal genetic map (VRH8771). Curves in plot indicate the genetic coordinate (x-axis) and LOD score (y-axis). The red dotted line represents the LOD significant threshold estimated with 1000 permutations for a level α of 0.05**


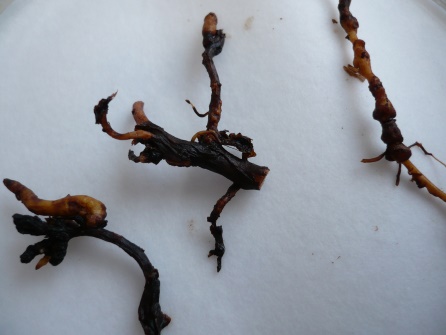


**Fig. S6 *In planta* experiment (A) illustration of the experimental device with each plant grown in an individual pot covered by a transparent plastic bell and (B) example of nodosities developed on roots (red arrows)**


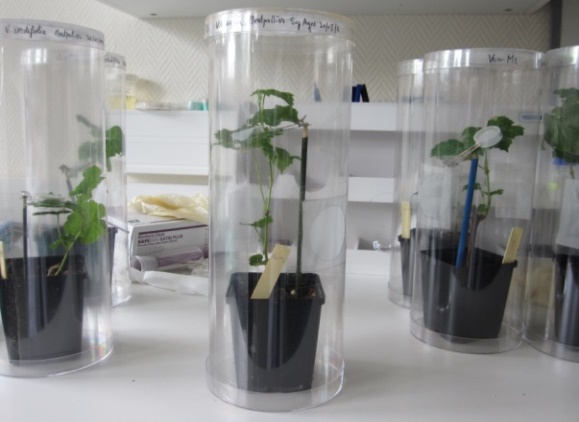

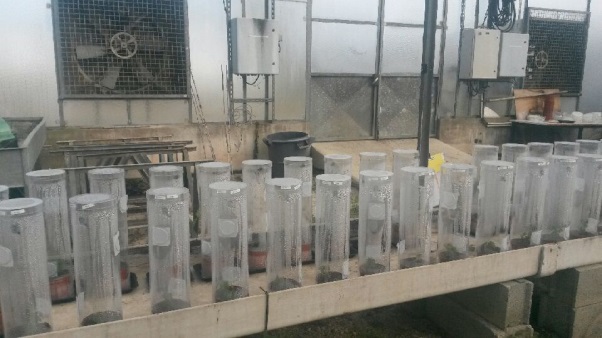


**A**

**B**
